# Asylum Administration

**Published:** 1897-02-20

**Authors:** 


### The Institutional Workshop.

### ASYLUM ADMINISTRATION.

### THE ROYAL INFIRMARY, EDINBURGH.

It is remarkable how small matters relating to great institutions have a tendency to assume an undue prominence. The Royal Infirmary goes on doing its beneficent work day by day, its wards swarming with students, and its physicians and surgeons successfully treating its thousands of patients. The wards and theatres have lately been brought up to the modern standard of sanitary and antiseptic conditions, while two new pavilions are being designed. Money is left to it in thousands year by year, and it is just going to ask for £70,000 more, and it will get it. The public of Scotland, rich and poor, believe in it as they do in their Bibles. But it is not faultless. There is no finality written on its portals. It has only one unchanging motto there—*Patet omnibus*.

All who have sat on the Boards of such institutions know that every now and again the routine of common administration is broken by new questions that cause some personal feeling, and then arise discussions that look at first as if they were serious. The fact is, human beings and institutions go in for periodic storms. It is dull work to meet week by week and have little to do but hear the minutes of the last meeting read. Such work does not satisfy the conscience of a Board. A "burning question"—or one that looked like it—has every now and then come up. Thus, for three or four years, "Should women students be admitted?" was keenly discussed and settled. "Should the staff of physicians and surgeons be represented by two or three of their number on its management?" has lately been the chief topic of excitement. There were always five or six medical men on the Board, nominated by the Royal Colleges and the University, some of these being usually old members of the staff. But the acting staff were not satisfied with this, and got the College of Surgeons to send to the board two of their number. It was affirmed and denied that this was according to the rules, and it was warmly argued, for and against, that there was involved a great principle in keeping distinct the governing and the executive of this institution. As it was time to revise the rules this year, this matter naturally came up for final settlement. The managers drew up a rule that any physician or surgeon of the infirmary, if appointed a manager, should *ipso facto* cease to be on the staff. The Court of Contributors, who have a veto in regard to all new rules, at their annual meeting in January upset this rule, and so the question was settled in a constitutional way.

No one can imagine that the infirmary would have suffered much which ever way the matter had ended. Good arguments were used for both sides of the question; yet one would think from some press comments that a tremendous conflict had taken place on which the usefulness and good management of this institution hung. One can see that in a question of this sort the doctors mainly took one side, and certain of the laity the other. Fortunately, the majority of the latter saw that it was not an unreasonable thing, and that it would be a gracious and appropriate thing to accede to the wishes of the distinguished men who gratuitously do all the medical work of the infirmary, and have made it the centre of the greatest medical school in the British Empire.

The Board of Management consists of large-minded, public-spirited men who, though differing, never for a moment quarrelled over so small a question. There has lately been some little talk about "undue economy" in the management. Not long ago the cry was the other way. In a public institution, spending from £40,000 to £50,000 a year of public money, there will always be abundant need for most strict inquiry into the outlays. Already a large saving has been effected thereby. The present energetic superintendent has worked hard to see that full value for every penny spent is got, the patients have in no way suffered, and the Board have backed him up in his zealous desire to further the interests of the institution. A new rule was added, about which there may be some question. Formerly the lady superintendent of nurses had the power to dismiss the junior nursing staff, subject to the approval of the general superintendent. The new rule provides that every dismissal of even the newest probationer must be reported to the House Committee before it takes effect. It was not alleged that the old rule had worked ill. But lady members had lately been added to the Board, and they strongly sympathised with the right of appeal. We trust the new rule may not weaken the discipline of the infirmary.

